# Pro-apoptotic activity of α-bisabolol in preclinical models of primary human acute leukemia cells

**DOI:** 10.1186/1479-5876-9-45

**Published:** 2011-04-21

**Authors:** Elisabetta Cavalieri, Antonella Rigo, Massimiliano Bonifacio, Alessandra Carcereri de Prati, Emanuele Guardalben, Christian Bergamini, Romana Fato, Giovanni Pizzolo, Hisanori Suzuki, Fabrizio Vinante

**Affiliations:** 1 Department of Sciences of Life and Reproduction, Section of Biochemistry, University of Verona, Italy; 2 Department of Medicine, Section of Hematology, University of Verona, Italy; 3 Department of Biochemistry "G. Moruzzi", University of Bologna, Italy

## Abstract

**Background:**

We previously demonstrated that the plant-derived agent α-bisabolol enters cells *via *lipid rafts, binds to the pro-apoptotic Bcl-2 family protein BID, and may induce apoptosis. Here we studied the activity of α-bisabolol in acute leukemia cells.

**Methods:**

We tested *ex vivo *blasts from 42 acute leukemias (14 Philadelphia-negative and 14 Philadelphia-positive B acute lymphoid leukemias, Ph^-^/Ph^+^B-ALL; 14 acute myeloid leukemias, AML) for their sensitivity to α-bisabolol in 24-hour dose-response assays. Concentrations and time were chosen based on CD34^+^, CD33^+^my and normal peripheral blood cell sensitivity to increasing α-bisabolol concentrations for up to 120 hours.

**Results:**

A clustering analysis of the sensitivity over 24 hours identified three clusters. Cluster 1 (14 ± 5 μM α-bisabolol IC_50_) included mainly Ph^-^B-ALL cells. AML cells were split into cluster 2 and 3 (45 ± 7 and 65 ± 5 μM IC_50_). Ph^+^B-ALL cells were scattered, but mainly grouped into cluster 2. All leukemias, including 3 imatinib-resistant cases, were eventually responsive, but a subset of B-ALL cells was fairly sensitive to low α-bisabolol concentrations. α-bisabolol acted as a pro-apoptotic agent *via *a direct damage to mitochondrial integrity, which was responsible for the decrease in NADH-supported state 3 respiration and the disruption of the mitochondrial membrane potential.

**Conclusion:**

Our study provides the first evidence that α-bisabolol is a pro-apoptotic agent for primary human acute leukemia cells.

## Background

**Figure 1:**
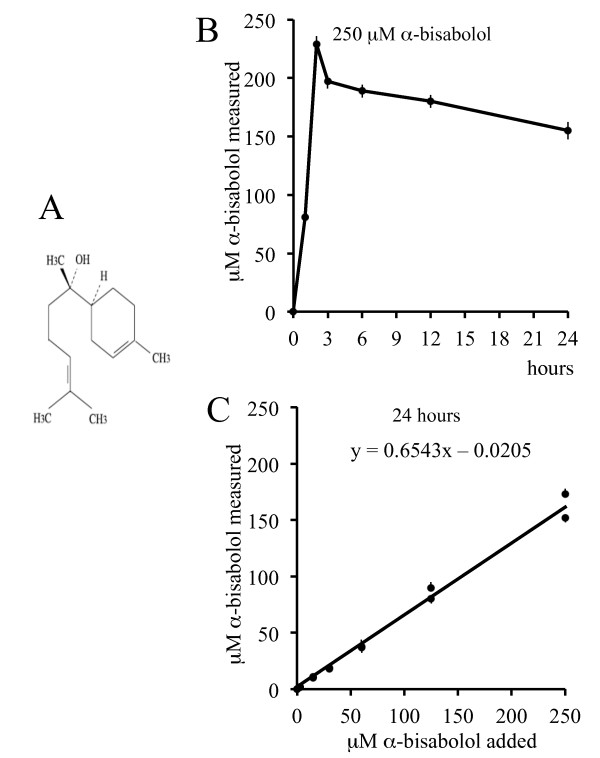
**α-bisabolol structure and solubilization in the culture medium**. (A) α-bisabolol is a small oily sesquiterpene alcohol with a molecular mass of 222.37 Da. (B) 250 μM α-bisabolol was added to culture medium: concentration raised during the first 3 hours, then lowered to around 65% of the initially added α-bisabolol after 24 hours. (C) By this time, the linear function relating added to measured concentrations of α-bisabolol shows that the incremental ratio was 0.65 for 14 evaluations representing a double series of 7 scaled concentrations tested by a RP-HPLC method. Each point is the mean ± SD of 2 measurements.

α-bisabolol is a small oily sesquiterpene alcohol (Figure 1A) that has been demonstrated to have activity against some malignant adherent human and rat cell lines [1] and against spontaneous mammary tumors in HER-2 transgenic mice [2]. We have previously found that it enters cells *via *lipid-rafts, interacts directly with BID, a pro-apoptotic BH3-only Bcl-2 family protein, and induces apoptosis [3].

Here we test the pro-apoptotic potential of α-bisabolol against primary acute leukemia cells, including Philadelphia-negative and -positive B acute lymphoid leukemias (Ph^-^/Ph^+^B-ALL) and acute myeloid leukemias (AML), and against normal blood white cells and hematopoietic bone marrow stem cells. Leukemic blasts represent a unique model to study the activity of α-bisabolol due to their biology allowing easy manipulation and evaluation. Moreover, acute leukemia treatment in adults is unsatisfactory despite investigations over the past four decades of a wide variety of anti-leukemic agents, refinement of bone marrow transplantation and the development of specific targeted therapy [4,5]. There is a particular need for treatments with both high efficacy and low toxicity [6] based on new molecules with mechanisms of action different from conventional drugs. This is especially true for elderly leukemia patients, who represent the majority of cases and have fewer therapeutic options [7]. Likewise, despite the introduction of anti-BCR/ABL tyrosine kinases for the treatment of Ph^+ ^leukemias, it seems that identification of novel compounds is perhaps necessary for success in eradicating Ph^+ ^cells [8,9].

The present study shows that α-bisabolol enters acute leukemic cells, where it disrupts the mitochondrial membrane potential and triggers apoptosis. Interestingly, α-bisabolol seems to be a much more effective agent in some Ph^-^B-ALL cells than in other types of acute leukemias at dosages that spare normal leukocytes and hematopoietic stem cells.

## Methods

### Patients and ethical requirements

Blasts from 28 patients with B-lineage ALL (14 Ph^-^, 14 Ph^+^B-ALL) and 14 with AML diagnosed at our institution, as well as blood and bone marrow cells from five healthy control donors, were collected after written informed consent was obtained, according to Italian law. All cellular studies were approved by the Verona University Hospital ethics committee. Patient characteristics are detailed in Table 1. The diagnosis of B-ALL or AML and their subtypes was based on clinical findings and on established morphological, cytochemical, cytofluorimetric, cytogenetic and molecular features of peripheral blood and bone marrow cells. AML patients received three induction courses according to standard AML treatment (1^st ^course: 3-day idarubicin + 7-day AraC by continuous i.v. infusion; 2^nd ^course: 3-day idarubicin + 3-day high-dose AraC; 3^rd ^course: 3-day high-dose AraC). B-ALL patients were treated with induction and maintenance therapy according to the VR95ALL protocol [10], which has been subsequently developed into the GIMEMA 0496 ALL protocol [11]. Young B-ALL patients (<18 years) were treated according to a specific pediatric protocol [12]. Ph^+^B-ALL patients underwent differential treatment including BCR/ABL TKI. Allogeneic bone marrow transplantation was performed during the first complete remission in four Ph^-^B-ALL cases and four Ph^+^B-ALL cases.

**Table 1:** Patients' characteristics.

patient	sex	age	diagnosis	Karyotype mol biol	therapy*	response^§^	relapse	OS^§§^
**Ph^- ^B-ALL**								
#01	M	22	B common	normal	1 + 2	CR	Yes	29+
#02	M	40	Pre-B	NA	1 + 2	CR	No	24+
#03	M	16	B common	normal	1	CR	Yes	12
#04	M	45	Pre-B	normal	1 + 2	CR	No	38+
#05	F	53	B common	hyperdiploid	1	CR	No	71+
#06	F	48	Pro-B	t(4;11)	1	CR	Yes	8
#07	F	42	Pro-B	t(4;11)	1	CR	Yes	6
#08	M	41	Pre-B	t(6;8)	1	CR	Yes	19
#09	M	59	Pro-B	t(4;11)	1	CR	Yes	10
#10	F	19	B common	hyperdiploid	1	CR	No	55+
#11	M	17	B common	t(17;22)	3	CR	No	9+
#12	F	53	B common	NA	1	CR	Yes	13+
#13	F	43	B common	normal	1 + 2	CR	Yes	25
#14	F	17	B common	normal	3	NR	Yes	4

**Ph^+ ^B-ALL**								
#01	M	44	Pre-B	Ph masked p210 (Y253H)	IM + D + 2	CR	No	12+
#02	F	54	B common	t(9;22) p210	1 (pre-IM)	no CR		36
#03	M	64	Pre-B	t(9;22) p210	IM	CHR, CCyR	Yes	9
#04	M	19	B common	t(9;22) NA (E255V)	1 + IM + N	no CHR		16
#05	M	40	Pre-B	t(9;22),-10 p210	1 + IM + D	CHR, CCyR	Yes	15
#06	F	38	Pre-B	t(9;22) p190 (T315I)	1 + IM + D	no CHR		9
#07	M	17	B common	t(9;22) p210	1 (pre-IM) + 2	CR	Yes	11
#08	M	70	Pre-B	t(9;22) NA	5 (pre-IM)	no CR		1
#09	M	35	B common	t(9;22), del(6) p190	1 + IM + 2	CCyR, MMR	No	46+
#10	M	63	B common	t(9;22) p190	IM + CS	CCyR, MMR	No	15+
#11	F	75	B common	hyperdiploid, t(9;22), NA	5 (pre-IM)	no CR		14
#12	M	89	Pre-B	t(9;22) p190	IM	CCyR, MMR	Yes	22+
#13	F	27	B common	t(9;22) p190	1 + IM	no CHR		10
#14	M	28	B common	t(9;22) p190	1 + IM + 2	CR	Yes	37

**AML**								
#01	M	59	M2	+4,+8	4	PR	Yes	7
#02	M	46	M0	NA	4	TD		1
#03	F	37	M4	del(X)(p21)	4	CR	No	167+
#04	F	47	M2	normal	4	NR	Yes	20
#05	F	70	M4 Eo	inv(16)	4	NR	no CR	6
#06	M	74	M4	normal	5			2
#07	M	62	M4	normal	4	NR	no CR	5
#08	M	69	M4 Eo	NA	5			5
#09	M	60	M2	-7	4	CR	No	38+
#10	M	83	M2	NA	5			3
#11	M	88	M2	NA	5			1
#12	F	79	M0	normal	5			9
#13	M	52	M4	normal	4	CR	No	24+
#14	F	61	M2	t(11;22)	4	CR	Yes	11

*Therapy: 1 = ALLVR589 protocol [10] or subsequent GIMEMA protocol LAL0496 [11]; 2 = allogeneic bone marrow transplantation; 3 = AIEOP-BFM-ALL 2000 protocol [12]; 4 = AML standard treatment (see Matherials and Methods)**; **5 = supportive care (hydroxicarbamide, blood transfusions etc); CS = corticosteroid; IM = imatinib; D = dasatinib; N = nilotinib

**^§^**NA = not avalaible; CR = complete remission; PR = partial remission; NR = non-responder; TD = toxic death; CHR = complete hematologic remission; CCyR = complete cytogenetic remission; MMR = major molecular remission (>3 log reduction bcr/abl ratio)

**^§§^**+ = ongoing follow-up

### Cells

#### 1. Primary Leukemic cells

Viable leukemic cells were purified by conventional methods from freshly heparinized peripheral blood with a circulating blast count ≥30,000/mL, or from full-substituted bone marrow that was frozen in liquid nitrogen at diagnosis [13]. In all cases frozen cell samples contained >95% blasts. Cell viability after thawing was always >90%, as assessed by trypan blue staining.

#### 2. Normal cells

Viable peripheral blood leukocytes [14] and bone marrow cells from - 4 - control donors were treated and used as specified above for leukemic cells.

#### 3. Cell line

The imatinib-sensitive BCR/ABL^+ ^CML-T1 cell line (T-lineage blast crisis of human chronic myeloid leukemia, purchased from DSMZ, Braunschweig, DE) was used to perform synergism studies.

### Measurement of α-bisabolol concentrations in the culture medium

α-bisabolol at a purity ≥95% (GC) was purchased from Sigma-Aldrich, St. Louis, MO. The dose-dependent solubilization of α-bisabolol in the culture medium over 24 hours was determined by a reverse-phase high performance liquid chromatography (RP-HPLC) method, developed in the Department of Food Science of Bologna University, Cesena office, Italy. All measurements were performed in duplicate. The α-bisabolol concentrations indicated throughout the article represent the calculated soluble fraction in the assay.

### Cytotoxicity assays

Cells derived from patients or normal donors were exposed for 24 hours to 20, 40, 80, and 160 μM α-bisabolol dissolved in ethanol (1:8 in order to minimize drug volumes), and when appropriate to 3 μM imatinib mesylate (Novartis, Basel, CH), representative of the in vivo active concentration. All cytotoxicity tests were performed in triplicate.

#### 1. Homogeneous cell populations

A lactate dehydrogenase (LDH) release assay was conducted as follows. Thawed cells were resuspended in RPMI-1640 (Lonza, Basel, CH) supplemented with 10% heat-inactivated fetal bovine serum (FBS, Lonza), 50 U/mL penicillin and 50 μg/mL streptomycin (complete medium, CM), seeded at a density of 2 × 10^6 ^cell/mL and incubated at 37°C in 5% CO_2_. After 24 hours, the cells were treated with α-bisabolol (or ethanol as a vehicle control) as specified above. Cytotoxicity was determined using the Cytotoxicity Detection Kit^PLUS ^according to the manufacturer's recommendations (Roche, Mannheim, DE). LDH leakage was measured as the ratio of treatment-induced LDH to spontaneous LDH release. α-bisabolol and imatinib mesylate data were reported as the percent cytotoxicity for treated compared to untreated cells and plotted as dose-response curves over 24 hours. The half maximal inhibitory concentration (IC_50_) was determined when appropriate.

#### 2. Heterogeneous cell populations

The absolute counts of normal leukocytes sub-populations were measured with TruCOUNT tubes (Becton Dickinson, San Jose, CA) by polychromatic flow cytometry according to the manufacturer's instructions with minor modifications. Peripheral blood and bone marrow cells were cultured with α-bisabolol for 24, 48, 72, 96 and 120 hours. At the end of the culture, 200 μL of sample, a mixture of antibodies (CD45 APC-H7, CD3 PE-Cy7, CD19 PE, CD14 APC for peripheral blood and CD45 APC-H7, CD34 PE, CD33 PE-Cy7 for bone marrow) and 7-amino-actinomycin D (all reagents from Becton Dickinson) for dead cells exclusion were added to the TruCOUNT tubes. After a 15-minute incubation at room temperature, 1 mL lysing reagent (Biosource, Nivelles, BE) was added for 10 minutes. A total of 40,000 beads were acquired on a FACSCanto cytometer (Becton Dickinson). A sequential Boolean gating strategy was used to accurately enumerate different populations [15].

### Cytotoxicity data hierarchical clustering analysis

To generate a classification based on α-bisabolol sensitivity, samples were grouped using the complete linkage hierarchical clustering algorithm available in the MultiExperiment Viewer (MeV, version 4.3 - http://www.tm4.org/mev/). A heat map for sensitivity was derived using the percentage data for mortality after adding α-bisabolol with respect to spontaneous mortality at the same time.

### Synergism studies

The interactions between imatinib mesylate and α-bisabolol were analyzed according to the median-effect method of Chou and Talalay [16] using the CalcuSyn Software (Biosoft, Cambridge, UK). The mean combination index (CI) values, based on constant drug ratios, were assessed with the following interpretation: CI>1, antagonistic effect; CI = 1, additive effect; CI<1, synergistic effect. Combination data were depicted as CI *vs*. fraction affected (Fa) plots, defining the CI variability by the sequential deletion analysis method. The cytotoxicity was evaluated as described above.

### Western blot analysis

Cells were homogenized at 4°C in 50 mM Tris-HCl (pH 8) containing 0.1% Nonidet-P40 (NP-40), 200 mM KCl, 2 mM MgCl_2_, 50 μM ZnCl_2_, 2 mM DTT, and protease inhibitors [1 mM phenylmethylsulfonyl fluoride (PMSF), 1 mg/mL leupeptin, and 1 mg/mL antipain]. Aliquots of the homogenates (40 μg total protein/lane) were loaded on SDS-polyacrylamide gels at the appropriate concentrations. Electrophoresis was performed at 100 V with a running buffer containing 0.25 M Tris-HCl (pH 8.3), 1.92 M glycine, and 1% SDS. The resolved proteins were electroblotted onto a nitrocellulose membrane using the iBlot™ system (Invitrogen, Carlsbad, CA). Membranes were then incubated with a mouse monoclonal IgG antibody to poly(ADP-ribose) polymerase (PARP) (Zymed, South San Francisco, CA), with a rabbit polyclonal IgG antibody to BID (Cell Signaling Technology, Danvers, MA) or with a rabbit polyclonal IgG antibody to α-tubulin (Cell Signaling Technology). The membranes were then washed and incubated with an anti-mouse or anti-rabbit IgG peroxidase-conjugated antibody (Cell Signaling Technology). The blots were washed again and then incubated with enhanced chemiluminescent detection reagents (Immun-Star™ WesternC™ Kit, Bio-Rad, Hercules, CA) according to the manufacturer's instructions. Proteins were detected using the ChemiDoc XRS Imaging System (Bio-Rad).

### Cytosolic and mitochondrial fraction preparation

Cell pellets were suspended in 100 μL of solution containing 10 mM NaCl, 1.5 mM MgCl_2_, 10 mM Tris-HCl, pH 7.5, 1 mM sodium orthovanadate, and complete EDTA-free protease inhibitor cocktail (Boehringer, Mannheim, DE). Cells were then chilled on ice for 10 minutes and gently lysed by adding 0.3% (v/v) NP-40. In order to restore an isotonic environment, a solution containing 525 mM mannitol, 175 mM sucrose, 12.5 mM Tris-HCl, pH 7.5, 2.5 mM EDTA, and protease inhibitor cocktail was added. Lysates were first centrifuged at 600 × *g *at 4°C in order to remove nuclei and then the supernatants were centrifugated at 17,000 × *g *for 30 minutes at 4°C. The obtained supernatants were collected and used as the cytosolic fraction. The pellets, that contained mitochondria, were washed once with the same buffer and then were resuspended in sample buffer. The cytosolic and the mitochondrial fractions were separated on a 15% SDS-PAGE and probed using a rabbit polyclonal IgG antibody to BID (Cell Signaling Technology). Then, the membrane with the cytosolic and mitochondrial fractions were probed with a rabbit polyclonal IgG antibody to α-tubulin (Cell Signaling Technology) and with a mouse monoclonal IgG antibody to Hsp60 (Abcam, Cambridge, UK), respectively.

### Cell permeabilization

Leukemic cells and normal lymphocytes were centrifuged (10 minutes, 200 × *g*) and washed with ice cold buffer A (250 mM sucrose, 20 mM HEPES, 10 mM MgCl_2 _- pH 7.1). The pellet was resuspended in 2 mL of buffer A containing 80 μg of digitonin. After a 1-minute incubation on ice, 8 mL of buffer A were added and cells were centrifuged (3 minutes, 400 × *g*). The pellet was resuspended in 100 μL buffer A containing 1 mM ADP, 2 mM KH_2_PO_3 _(respiration buffer) and immediately used for the polarographic assay. Cell number and permeabilization was measured by the trypan blue exclusion method.

### Oxygen consumption

Permeabilized leukemic cells and lymphocytes were assayed for oxygen consumption at 30°C using a thermostatically controlled oxygraph and Clark electrode. Cells were incubated for 10 minutes in respiration buffer at 30°C in the presence or absence of 3 μM α-bisabolol. Mitochondrial respiration (state 3 respiration) was started by adding 5 mM glutamate plus malate (G/M) and 5 mM succinate plus glycerol-3-phosphate (S/G3P), which are complex I and complex III/glycerol-3-phosphate dehydrogenase substrates, respectively. The maximal respiration rate (uncoupled respiration) was empirically determined by the addition of 200 nM carbonylcyanide-4- (trifluoromethoxy)-phenylhydrazone (FCCP). Oxygen consumption was completely inhibited by adding 4 μM antimycin A at the end of the experiments [17].

### Mitochondrial membrane potential evaluation

Cells resuspended in CM at 1 × 10^6^/mL were treated with 40 μM α-bisabolol for 3 or 5 hours at 37°C. They were then washed with pre-warmed CM, 4 μM of the potential sensitive dye JC-1 (5,5',6,6'-tetra-chloro-1,1',3,3'-tetra-ethyl-benz-imidazolyl-carbocyanine iodide, Molecular Probes, Eugene, OR) was added, and they were then placed back into the incubator. After 30 minutes they were washed twice with pre-warmed PBS. An aliquot of each sample was spotted onto a slide, mounted with a coverslip and immediately recorded by an Axio Observer inverted microscope (Zeiss, Gottingen, DE). Visualization of JC-1 monomers (green fluorescence) and JC-1 aggregates (red fluorescence) was done using filter sets for fluorescein and rhodamine dyes (emission 488 and 550 nm respectively). Image captures of random fields using fixed imaging parameters were performed, and previously unviewed areas of cells were captured to avoid photobleaching [18]. Image analysis was done using Axiovision 3 software. The other aliquot of each sample was resuspended in PBS and analyzed using a FACSCalibur cytometer (Becton Dickinson) equipped with a 488 nm argon laser. The emission of JC-1 monomers was detected in Fl-1 using a 530/30 nm bandpass filter, and JC-1 aggregates were detected in Fl-2 using a 585/42 nm bandpass filter. FlowJo 8.8.2 software (Tree Star, Ashland, OR) was used to analyze data [19].

### DNA ladder

For internucleosomal DNA laddering analysis, 5 × 10^6 ^cells were resuspended in 0.3 mL of culture medium containing 10% FBS and incubated for 90 minutes at 65°C and then overnight at 37°C in the presence of 0.4 M NaCl, 5 mM Tris-HCl (pH 8), 2 mM EDTA, 4% SDS and 2 mg/mL proteinase K. The lysates were brought to a final concentration of 1.58 M NaCl and centrifuged twice for 10 minutes at 6,000 × *g *to separate the DNA fragments from intact DNA. The supernatants were recovered, and DNA was precipitated by the addition of three volumes of absolute ethanol at -80°C for 1 hour. The DNA pellets were recovered by microcentrifugation (10 minutes, 12,000 × *g*) and resuspended in a minimal volume of 40 μl of 10 mM Tris-HCl (pH 7.4), 1 mM EDTA, and 1 mg/mL DNase-free ribonuclease A. Aliquots of 5 μg of DNA were then loaded onto a 1% agarose gel containing 0.25 μg/mL ethidium bromide. After electrophoresis, the DNA was visualized by UV light using the ChemiDoc XRS Imaging System (Bio-Rad).

### Statistics

Student's t-test for means, chi-squared tests, Mann-Whitney U test and Kruskall-Wallis analysis of variance by ranks were considered significant for *p *values < 0.05. The 24-hour IC_50 _was approximated by using mean cytotoxicity data in the different groups (according to diagnosis or clustering-based analysis).

## Results

### α-bisabolol concentrations in the culture medium

Due to the lipophilic properties of α-bisabolol, a preliminary evaluation was performed of the dose-dependent solubilization in the culture medium over 24 hours by a RP-HPLC method. The addition of α-bisabolol at time 0 was followed by a rapid increase of the measured concentrations during the first 3 hours. After 24 hours, concentrations may be considered roughly constant, though with a slightly downward trend (Figure 1B). A double series of 7 determinations corresponding to 0, 3, 15, 30, 60, 125, 250 μM α-bisabolol added to medium gave a linear function with a 0.65 incremental ratio (Figure 1C), indicating that, after 24 hours, around 65% of the α-bisabolol added was actually measured in the culture medium.

### α-bisabolol cytotoxicity in normal peripheral blood cells

**Figure 2:**
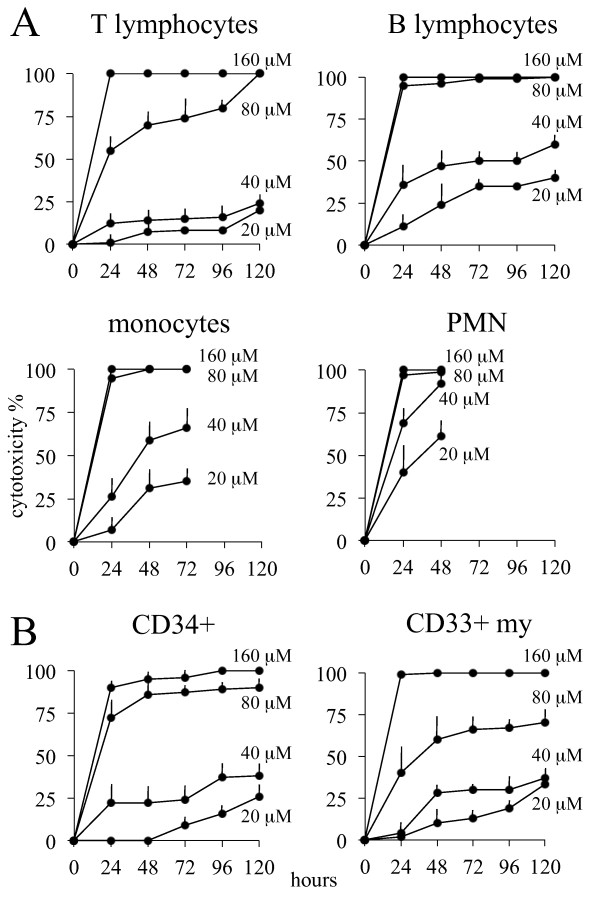
**Cytotoxicity of α-bisabolol in normal hematologic cells**. (A) Peripheral blood cells. (B) Bone marrow stem cells. Time- and dose-response curves between 20 and 160 μM α -bisabolol in the 120-hour cytotoxicity assays. Means ± SD of 5 normal donors are depicted.

The viability of normal blood cells was evaluated after different times and doses of exposure to α-bisabolol. The cytotoxicity increased in a dose- and time-dependent manner. Figure 2A depicts the sensitivity to increasing doses of α-bisabolol for up to 120 hours in each different blood cell subpopulation. T lymphocytes, which were far less sensitive to α−bisabolol than B-lymphocytes, monocytes and neutrophils, had a 24-hour IC_50 _of 59 ± 7 μM and were only marginally sensitive to 40 μM α-bisabolol over 120 hours.

### α-bisabolol cytotoxicity in normal counterparts of acute leukemia cells

Figure 2B depicts the sensitivity to α-bisabolol in CD33^+^my and CD34^+^/33^+ ^or CD34^+^/19^+ ^cells from 5 normal bone marrow samples. These subpopulations were assumed to represent the normal counterpart of acute leukemia blasts and the hematopoietic compartment that is responsible for bone marrow renewal and, eventually, drug toxicity. The 24-hour α-bisabolol IC_50 _was 95 ± 7 and 62 ± 9 μM in CD33^+^my and CD34^+ ^cells, respectively (*p *< 0.05). By contrast, no difference was observed between CD34^+^/33^+ ^and CD34^+^/19^+ ^cells (64 ± 6 and 63 ± 4 μM IC_50_, respectively).

### α-bisabolol cytotoxicity in primary acute leukemia cells by diagnosis

**Figure 3:**
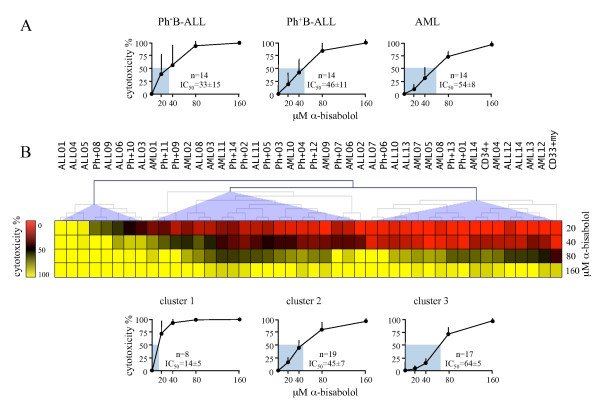
**24-hourcytotoxicity of α-bisabolol in primary blasts from 42 acute leukemias**. (A) α-bisabolol activity against blasts *ex vivo*, here grouped by diagnosis. The corresponding IC_50 _values and number of cases (n) are shown. The differences between the Ph^-^B-ALL sensitivity curves and the other ones were statistically significant (*p *< 0.05). Each point is the mean ± SD of 14 cases. (B) α-bisabolol sensitivity clustering analysis. The samples were grouped by complete linkage hierarchical clustering algorithm available in MultiExperiment Viewer http://www.tm4.org/mev/. The heat map was obtained by subtracting spontaneous mortality to scaled α-bisabolol 24-hour cytotoxicity expressed as percentage. Three main groups of patients were identified based on their cytotoxicity response. The Ph^-^B-ALL (ALL) cases shared the highest sensitivity and were grouped mainly in the first sensitivity cluster, whereas AML cases were split into two groups with intermediate and lower sensitivities. Ph^+^B-ALL (Ph+) cells were scattered among the three groups, although they were mainly clustered in the second group. At the bottom, the 24-hour dose-response curves of the three sensitivity clusters are depicted, and the corresponding IC_50 _values and number of cases (n) are shown. The differences between the curves were statistically significant (*p *< 0.05).

Based on these data from normal cells, we performed *ex vivo *dose-response (20, 40 80, and 160 μM α-bisabolol) cytotoxicity assays at 24 hours in 42 different samples of leukemic cells (14 Ph^-^B-ALL, 14 Ph^+^B-ALL, 14 AML) obtained from patients before any treatment. Table 1 summarizes the main patients' characteristics. Table 2 shows the results of the cytoxicity assays as mean ± SD after 24 hours of exposure to different concentrations of α-bisabolol, and Figure 3A depicts the corresponding dose-response curves for Ph^-^B-ALL, Ph^+^B-ALL, and AML cells. The 24-hour dose-response assays showed that α-bisabolol was cytotoxic to primary Ph^-^B-ALL cells (33 ± 15 μM IC_50_). Though less sensitive, Ph^+^B-ALL, including Ph^+^-cells resistant to imatinib mesylate, and AML cells were also killed (46 ± 11 and 54 ± 8 μM IC_50_, respectively; *p *< 0.05 compared to Ph^-^ALL). Thus, α-bisabolol is a pro-apoptotic agent for acute leukemia cells *ex vivo*, particularly for Ph^-^B-ALL.

**Table 2:** α-bisabolol cytotoxicity in acute leukemia cells and in their normal counterparts (% mean values ** ± **SD according to α-bisabolol concentration).

μ**M **α**-bisabolol**		20	40	80	160	IC_50_	*p*
Ph^-^B-ALL	14	37 ± 36	55 ± 37	87 ± 12	100	33 ± 15	***<0.05*** ***ns***
Ph^+^B-ALL	14	2 ± 19	42 ± 28	81 ± 17	98 ± 2	46 ± 11	
AML	14	10 ± 9	32 ± 21	72 ± 11	96 ± 4	54 ± 8	

cluster 1	8	72 ± 24	94 ± 6	99 ± 1	100	14 ± 5	***<0.05*** ***<0.05***
cluster 2	19	17 ± 9	44 ± 14	77 ± 15	97 ± 3	45 ± 7	
cluster 3	15*	3 ± 3	14 ± 8	73 ± 14	98 ± 2	65 ± 5	
	17^§^	2 ± 3	14 ± 8	71 ± 16	97 ± 3	64 ± 5	

CD34^+^	5	1 ± 2	22 ± 19	72 ± 35	98 ± 1	62 ± 9	***ns*** ***<0.05***
CD33^+^my	5	2 ± 2	4 ± 3	40 ± 39	99 ± 1	95 ± 7	

*acute leukemia samples

^§^15 acute leukemias plus CD34^+ ^and CD33^+^my samples

### α-bisabolol cytotoxicity on primary leukemic cells by clustering analysis

**Figure 4:**
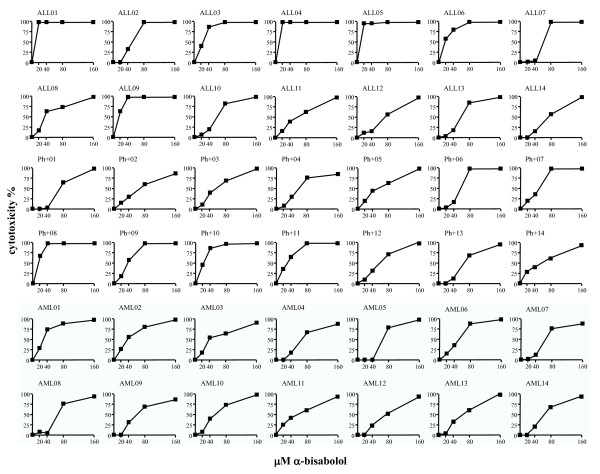
**24-hour cytotoxicity of α-bisabolol in each individual case**. 14 Ph^-^B-ALL, 14 Ph^+^B-ALL, and 14 AML cell samples were treated with 20, 40, 80, and 160 μM α-bisabolol over 24 hours. Captions identify the cases in Table 1 and in Figure 3B (clustering analysis).

We generated a cytotoxicity-based classification of our leukemic samples using the complete linkage hierarchical clustering algorithm available in MultiExperiment Viewer. As shown in Figure 3B, clustering analysis identified three main groups (*p *< 0.05) by comparing differences among experimental samples with regard to responsiveness to apoptotic signals induced by α-bisabolol. The group with the highest sensitivity to α-bisabolol (cluster 1: 14 ± 5 μM IC_50_) included 2 Ph^+ ^and 6 Ph^- ^B-ALL cases. Thus, a proportion of the Ph^-^B-ALL cases shared a high sensitivity to α-bisabolol, although some other Ph^-^B-ALL were scattered over different sensitivity groups. The AML cases were split into two groups with intermediate (cluster 2: 45 ± 7 μM IC_50_; 7 AML cases) and lower (cluster 3: 65 ± 5 μM IC_50_; 7 AML cases) sensitivity. Unlike Ph^-^B-ALL, AML cases as a whole were less sensitive to α-bisabolol. The Ph^+^B-ALL cases were scattered all over the three groups but were mainly clustered with intermediate sensitivity AML. Interestingly, introducing both CD34^+ ^and CD33^+^my cell sensitivity to α-bisabolol (as the mean value of 5 cases) in clustering analysis made it evident that ALL cells as a whole were more sensitive to α-bisabolol than their normal counterpart (grouped into cluster 3 among less sensitive cells). This analysis demonstrated that some Ph^-^B-ALL cases may be highly sensitive to the apoptotic mechanisms activated by α-bisabolol and indicated that the Ph^+^B-ALL cases and especially the AML cases (these latter showing a bimodal sensitivity) may well be characterized by variable degrees of resistance to these mechanisms. Still, all leukemia cases were eventually responsive to 65 μM α-bisabolol for 24 hours (Table 2). In Figure 4, the dose-response assays for each case are depicted.

### α-bisabolol plus imatinib mesylate in cells bearing mutated or non-mutated BCR/ABL

**Figure 5:**
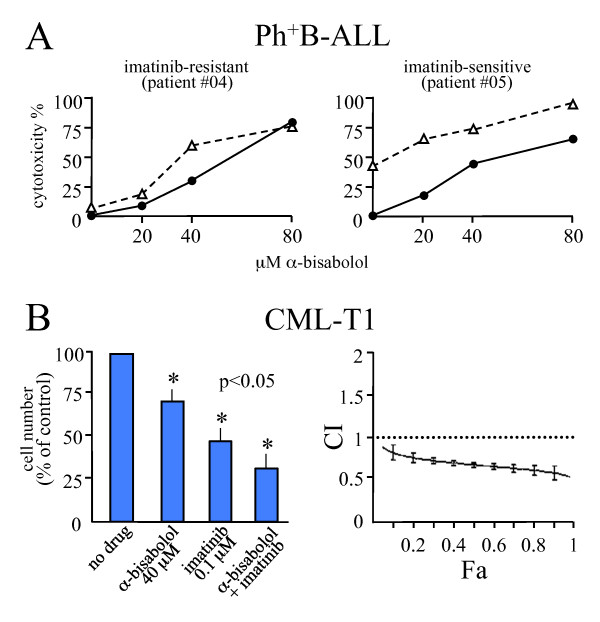
**24-hour cytotoxicity of α-bisabolol in Ph^+ ^cells as compared to imatinib mesylate**. (A) Scaled α-bisabolol alone (solid line) or in combination with 3 μM imatinib mesylate (dashed line) in 2 representative cases out of 10 (Ph^+^B-ALL #04 and #05 in Table 1, Figure 3B and Figure 4, where α-bisabolol concentrations are represented up to 160 μM). The imatinib mesylate-dependent cytotoxicity is indicated at point 0,0. Cells resistant to imatinib mesylate were sensitive to α-bisabolol. In cells sensitive to imatinib mesylate, α-bisabolol potentiated the effect of the other drug. (B) Analysis of synergism between imatinib and α-bisabolol in the imatinib-sensitive BCR/ABL^+ ^human cell line CML-T1. *Left side*. Effects of 40 μM α-bisabolol and 0.1 μM imatinib, alone and combined, on proliferation of the cell line. Means ± SD of 5 experiments. *Right side*. Plot showing the corrisponding combination index (CI) *vs*. the fraction affected (Fa). CI values are <1, indicating that the two drugs are synergistic. Bars represent the variability of effects according to the sequential deletion analysis [16].

α-bisabolol was active against Ph^+^B-ALL cells (24-hour IC_50 _was 46 ± 11 μM; Figure 3). We wondered if α-bisabolol and imatinib mesylate had synergistic effects. As shown in Figure 5A cells from case Ph^+^B-ALL #04 (carrying the E255V mutation, Table 1) were primarily resistant to imatinib mesylate and showed similar *ex vivo *cytotoxicity when treated with either α-bisabolol (20, 40 80, and 160 μM for 24 hours) alone or α-bisabolol associated with imatinib mesylate (3 μM for 24 hours representative of *in vivo *effective concentration). In contrast, cells sensitive to imatinib mesylate shared a significant increase in cytotoxicity to α-bisabolol. For instance, cells from patient Ph^+^B-ALL #05 (Table 1) shifted from 40% cytotoxicity with 40 μM α-bisabolol alone to 75% with α-bisabolol plus imatinib mesylate. This may suggest that the presence of BCR/ABL tyrosine kinase activity in a cell reduces the effectiveness of α-bisabolol as a pro-apoptotic agent or that imatinib mesylate reduces the IC_50 _of α-bisabolol. The imatinib mesylate-sensitive BCR/ABL^+ ^CML-T1 cell line, a T-cell lineage blast crisis of CML, was used in order to conclusively calculate the synergism, if any, between imatinib mesylate and α-bisabolol. Figure 5B shows that the combination of imatinib mesylate and α-bisabolol resulted in a higher degree of inhibition of cellular proliferation compared with each inhibitor alone (*p *< 0.05), and the combination was clearly synergistic, denoted by CI values <1 for any given Fa [16]. Also, the combination resulted in a higher degree of induction of apoptosis (data not shown).

### α-bisabolol and BID

**Figure 6:**
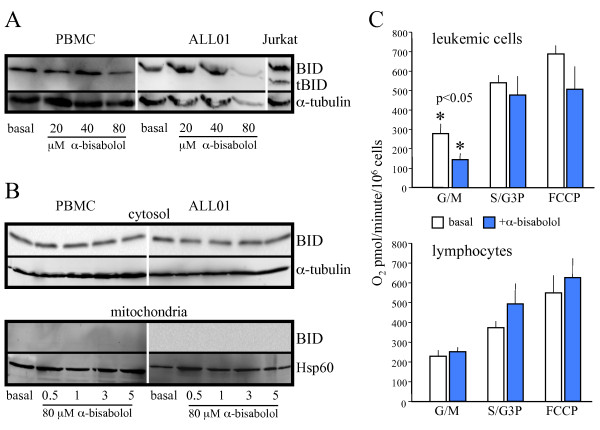
**BID and NADH-supported state 3 respiration in normal PBMCs and leukemic blasts treated with α-bisabolol**. (A) 24-hour α-bisabolol did not induced the cleavage of BID (full length 22 kDa, cleaved 15 kDa) at any concentration. Etoposide-treated Jurkat cells were used as a positive control for tBID. (B) No BID translocation was detected in mitochondrial fraction at different times and solubilized doses of α-bisabolol. α-tubulin and Hsp60 were used as markers for the cytosol and mitochondria fractions, respectively. A representative case is shown. (C) Permeabilized leukemic cells and healthy lymphocytes were incubated for 10 minutes in respiration buffer at 30°C in the presence or in the absence of 3 μM α-bisabolol. In treated leukemic cells, the G/M oxygen consumption was clearly lower than in untreated leukemic controls (*p *< 0.05). The S/G3P oxygen consumption was not modified by treatment, and the mitochondrial respiration was not stimulated by FCCP addition. This is in line with a direct effect of α-bisabolol on mitochondrial integrity. Healthy lymphocyte respiration was not affected by treatment. G/M: glutamate plus malate; S/G3P: succinate plus glycerol-3-phosphate; FCCP: carbonylcyanide-4-(trifluoromethoxy)-phenyl-hydrazone. Means ± SD of 6 leukemias and 6 normal donors are depicted.

We have previously demonstrated that α-bisabolol binds to the BCL-2 family member BID [3]. To evaluate the possibility that the treatment with α-bisabolol leads to the cleavage of BID to truncated BID, we analyzed whole extract of leukemic cells and normal PBMCs by Western blot. As shown in Figure 6A, whereas truncated BID is detectable in the human T-cell lymphoblast-like cell line Jurkat used as a positive control, it is not present in PBMCs and blasts, indicating that the pro-apoptotic action of α-bisabolol is not dependent on BID cleavage. However, caspase cleavage is not an absolute requirement for activating BID pro-apoptotic function. Full-length BID is also capable of translocation to the mitochondria, where it has been shown to potentiate cell death following certain apoptotic signals [20]. But we were unable to demonstrate full-length BID in the mitochondria by separating cytosolic and mitochondrial fraction following α-bisabolol treatment (Figure 6B).

### Decrease of mitochondrial state 3 respiration

In a previous paper, we confirmed the mitochondrial involvement in α-bisabolol-induced cell death by the measurement of oxygen consumption by intact cells [21]. In the current work we used permeabilized leukemic cells from 6 patients (3 Ph^-^B-ALL, 1 Ph^+^B-ALL, 2AML) and healthy lymphocytes from 6 donors to determine whether α-bisabolol treatment affects mitochondrial state 3 and uncoupled respiration. Figure 6C shows that NADH-supported state 3 respiration (G/M) in α-bisabolol-treated leukemic cells was dramatically decreased in comparison with untreated leukemic controls (140.0 ± 70.5 *vs*. 280.7 ± 11.9 pmol O_2_/minute/10^6 ^cells; *p *< 0.05). In contrast, the oxygen consumption sustained by S/G3P oxidation was not affected by α-bisabolol treatment, and the mitochondrial respiration was not stimulated by the addition of FCCP. These data are in line with a loss of mitochondrial integrity in treated leukemic samples, which is responsible for the matrix NADH decrease. This behavior is confirmed by the observation that the respiration in the presence of S/G3P was unaffected. Healthy lymphocyte respiration was not statistically modified by α-bisabolol treatment in state 3 using G/M and S/G3P as substrates and FCCP as a mitochondrial uncoupler. This is in agreement with the resistance to α-bisabolol observed in lymphocytes (Figure 2A).

### Loss of mitochondrial potential

**Figure 7:**
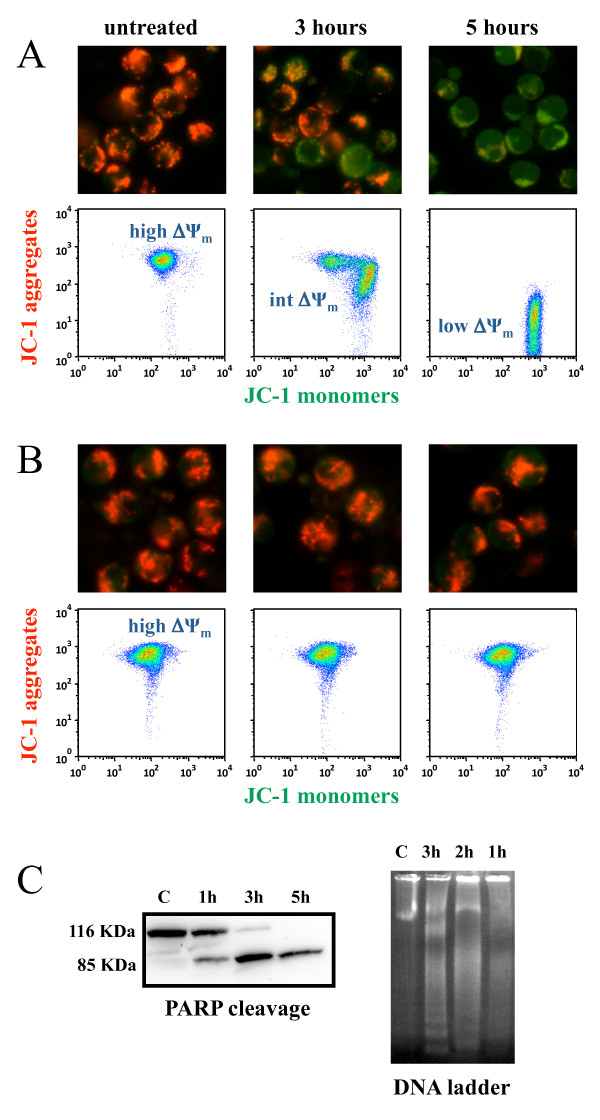
**α-bisabolol-induced mitochondrial damage in primary leukemic blasts**. Cells were stained with JC-1. In non-damaged cells, JC-1 forms red-emitting aggregates in the mitochondrial matrix. A loss of red fluorescence and an increase in cytoplasmic green-emitting monomers signal the disruption of the mitochondrial transmembrane potential (ΔΨm). (A) The representative case Ph^-^B-ALL #01 is shown out of the 6 leukemias tested. *Microscopy (magnification, × 400)*. Whereas untreated leukemic blasts showed well-polarized mitochondria marked by punctated red fluorescent staining, blasts treated with 40 μM α-bisabolol had staining that was quite completely replaced by diffuse green fluorescence, indicating loss of ΔΨm. *Flow cytometry*. Untreated blasts with well-polarized mitochondria localized in the upper region of the plot (high ΔΨm). Blasts exposed to 40 μM α-bisabolol shifted right and downward (intermediate and low ΔΨm), due to the progressive dislocation of JC-1 from the mitochondria to the cytoplasm, which signaled the disruption of the mitochondrial ΔΨm. (B) Both untreated and α-bisabolol-treated normal lymphocytes used as a negative control maintained well-polarized mitochondria and did not undergo apoptosis. Apoptosis of leukemic blasts was also documented by (C) PARP cleavage and DNA laddering in the same representative case depicted in (A).

JC-1 staining [19,22] demonstrated that α-bisabolol dissipates the mitochondrial transmembrane potential (ΔΨ_m_). In fit cells, JC-1 is more concentrated in the mitochondria (driven there by the ΔΨ_m_), where it forms red-emitting aggregates, than in the cytosol, where it exists as a green-fluorescent monomer. Accordingly, the ratio red/green JC-1 fluorescence can be used as a sensitive measure of ΔΨ_m _[23]. Disruption of ΔΨm (a hallmark of cytochrome *c *translocation and the start of the apoptotic process) is indicated by a loss of red fluorescence and an increase in green fluorescence. Figure 7A shows the representative case Ph^-^B-ALL #01 out of the 6 tested. Microscopy revealed that in untreated leukemic cells well-polarized mitochondria were marked by punctate red fluorescent staining (Figure 7A, left side). After a 3-hour incubation with 40 μM α-bisabolol, this pattern was replaced by diffuse green fluorescence in leukemic cells (Figure 7A, center and right side). Flow cytometry showed that untreated blasts with well-polarized, red-emitting mitochondria localized in the upper region of the plot (Figure 7A, left plot: high ΔΨ_m_). Blasts exposed to 40 μM α-bisabolol underwent a progressive loss of red fluorescence, indicated by a shift right and downward over 3 (Figure 7A, central plot: intermediate ΔΨ_m_) and 5 hours (Figure 7A, right plot: low ΔΨ_m_). In contrast, normal lymphocytes used as a negative control did not suffer any changes in their microscopy or cytofluorimetric pattern when exposed to a similar α-bisabolol concentration, indicating that there was no mitochondrial damage (Figure 7B, images and plots), and that the cells remained vital. Finally, the same blasts depicted in Figure 7A underwent PARP cleavage and DNA laddering following α-bisabolol exposure (Figure 7C).

## Discussion

Forecasting the fraction of the lipophilic compound α-bisabolol that was dissolved in water at given times was a basic preliminary step to standardize the drug use in the conditions of our cytotoxicity tests. In these conditions, we and an independent analytical laboratory repeatedly measured that after 24 hours in culture medium α-bisabolol lowered to around 65% of the theoretical concentrations added. In contrast, we could obtain only a 2.5% solubility fraction in previous studies [1]. Thus, the present experiments led to define conditions of maximal water solubility for α-bisabolol.

By cluster analysis, we separated out three subgroups of leukemias with different sensitivities over 24 hours. α-bisabolol was effective with an IC_50 _of 14 ± 5 μM in a substantial proportion of Ph^-^B-ALL, and with an IC_50 _of 45 ± 7 μM in a substantial proportion of both Ph^+^B-ALL and AML cases. Remarkably, these concentrations spared normal circulating leukocytes and CD34^+ ^and CD33^+^my hematopoietic bone marrow precursors. HUVECs, fibroblasts and hepatocytes were also spared (personal observation). The third subgroup included mainly, but not exclusively, AML samples with an IC_50 _value of 65 ± 5 μM. Thus, Ph^-^B-ALL cases were definitely more sensitive than AML cases, whose IC_50 _was near to that observed *in vitro *also in normal leukocytes, except lymphocytes, and in hematopoietic precursors. Nevertheless, previous studies in animal models suggested that similar α-bisabolol concentrations may be safely administered through daily oral supplementation even on a long-term basis [24,25]. The α-bisabolol concentrations that we found active against leukemic cells *in vitro *are also lower than, or similar to, the concentrations that we measured in the blood and in the brains of healthy mice sacrificed after treatment with 1.4 g/Kg α-bisabolol. In these mice the blood parameters of liver and kidney fucntionality were preserved and, remarkably, the concentration in the brain exceeded 50 μM without toxicity. Therefore, an active concentration of α-bisabolol safely accumulated in a body environment where lymphoid blasts have a tendency to localize and survive protected from a number of curative drugs [26]. A dose of 10 mg/mouse α-bisabolol induced a decrease in the number of palpable mammary tumor masses without adverse reaction in HER-2 transgenic mice [2].

Ph^+^B-ALL cells were also sensitive to α-bisabolol. In three cases (Ph^+^B-ALL #01, #04, #06 in Table 1) with primary mutation of BCR/ABL, we observed a full efficacy of α-bisabolol. In imatinib mesylate-sensitive blasts, the association of α-bisabolol and imatinib mesylate led to a synergistic effect which we have conclusively calculated as a CI<1 at any given Fa [16] in the BCR/ABL^+ ^human cell line CML-T1. It is not clear, however, whether the synergism depends on internalization mechanics or on intracellular modulation of the damaging actions of each or both drugs. A compound like α-bisabolol - and others [27] - could help to identify profitable new strategies for both mutated and non-mutated leukemias [9,28,29].

Our biochemical data suggest a direct effect on mitochondrial integrity as a possible mechanism of α-bisabolol damage to leukemic cells. This behavior is supported by the observed oxygen consumption decrease in the presence of glutamate/malate and by the unaffected respiration rates in the presence of succinate/glycerol-3-phosphate. Microscopy and flow cytometry data show that α-bisabolol disrupts ΔΨ_m_, which induces outer membrane permeabilization and leads to the apoptotic death of blasts. Our data not only implicate α-bisabolol for the first time in mitochondrial impairment in human leukemic cells but also suggest that this goes through a peculiar model of cell death, i.e., the formation of a cellular population with intermediate DΨ _m _which is a feature of apoptosis seen only in a few cell types and never described to date in leukemic blasts [30].

In all leukemia samples treated with α-bisabolol, BID was found to be expressed in a full-lenght form that was suitable for binding to α-bisabolol. We failed to demonstrate full-length BID translocation to the mitochondria in leukemic cells as a pro-apoptotic mechanism [19]. Nevertheless, BID might act as a carrier that conveys α-bisabolol to the mitochondrial membrane.

Thus, according to our previous and present work, α-bisabolol enters cells *via *lipid rafts and directly involves mitochondrial permeability transition pore opening [20], which is responsible for the reduced glutamate/malate-supported oxygen consumption and leads to disruption of the mitochondrial membrane potential and programmed cell death. The reciprocal role of BID and α-bisabolol [3] remains elusive in leukemic cells.

## Conclusion

We provide here the first evidence that α-bisabolol is an effective pro-apoptotic agent in primary ALL cells at concentrations and durations that spare normal blood and bone marrow cells. It retains cytotoxic potential in both imatinib mesylate-resistant and -sensitive Ph^+^B-ALL. It is also active against primary AML cells at slightly higher concentrations. Our findings support α-bisabolol as a possible candidate for the treatment of acute leukemias and establish a basis for studies in animal models.

## Competing interests

The authors declare that they have no competing interests.

## Authors' contributions

EC, AR, ACdP performed the research, analyzed data, and performed statistical analysis; MB, EG, CB, RF contributed analytical tools, performed selected experiments and analyzed data; GP contributed criticism; HS suggested the research, contributed ideas and critical scientific knowledge, analyzed and interpreted data; FV chose the clinical setting, designed and performed the research, analyzed and interpreted data, and wrote the paper; all authors checked the final version of the manuscript.
